# Response of drought index to land use types in the Loess Plateau of Shaanxi, China

**DOI:** 10.1038/s41598-022-12701-8

**Published:** 2022-05-23

**Authors:** Weixuan Wang, Chenfeng Cui, Weihua Yu, Liang Lu

**Affiliations:** 1 College of Water Resources and Architectural Engineering, Northwest A&F University, Yangling, 712100 Shaanxi China; 2 The Key Laboratory of Agricultural Soil and Water Engineering in Arid Areas, Ministry of China, Northwest A&F University, Yangling, 712100 Shaanxi China

**Keywords:** Climate sciences, Hydrology, Natural hazards

## Abstract

Drought is influenced by numerous factors, and traditional studies have only considered meteorological factors, but human activities are also an important influencing factor. From 1980 to 2010, the reform and opening up and the project of returning farmland to forest have largely changed the land use type of Loess Plateau in Shaanxi. In order to study the influence of land use types on drought in the study area, 8 stations with different land use types were selected based on remote sensing data and meteorological data. Based on univariate linear regression, the response of the drought index SPI to land use types was analyzed sequentially in each of the five time stages. The results showed that from 1980 to 2000, forest had the greatest drought intensity, followed by arable land and town and country. However, the response of arable land to the drought index was the greatest, followed by town and country. Forest had the weakest response to the drought index. From 2000 to 2010, the greatest degree of drought was observed in forest, followed by town and country and arable land. And forest has the strongest response to the drought index, followed by town and country, and finally, arable land. The area of forest, grass and town and country keeps increasing. The area of arable land is decreasing year by year. Land use types are constantly undergoing complex transformations. The drought index responds strongly to the change of both arable land to grass and arable land to town and country, while it responds weakly to the mutual transformation of both grass and town and country. In the areas where meteorological conditions are difficult to change, the local drought is considered to be improved by changing the substrate type.

## Introduction

Drought is a kind of natural disaster which is common and widespread, with high frequency, long time span, wide impact and profound influence on agriculture and economy. In China, drought losses account for more than 15% of natural disasters. The area of drought is as high as 57% of the total area affected by natural disasters. The frequency of droughts accounts for about 1/3 of the total frequency of disasters^1^. Drought affects four major geographical aspects: meteorology, hydrology, agriculture, and social economy. The classification methods have reached a consensus^2^. Since long-term meteorological drought will form soil and hydrological drought, and long-term soil and hydrological drought will lead to agricultural drought, meteorological drought indicators are particularly important for drought monitoring^3^. Therefore, the study of meteorological drought has extremely important practical significance.

In meteorological drought, the drought index is an important indicator based on meteorological and hydrological variables that can reflect different aspects of drought. As the most commonly used meteorological drought monitoring index, the standardized rainfall index has a good performance^4^. SPI can better reflect the intensity and duration of drought, is more sensitive to drought changes, and at the same time the characteristics of multi-time scale application can serve for drought monitoring at different time scales^5^. Sun et al. used SPI to study the drought characteristics in the Loess Plateau region of Shaanxi and found that SPI has good applicability in Shaanxi^6^. Therefore SPI was selected as a monitoring indicator in this study. However, drought is influenced by numerous factors. Climate change, human activities, and surface water system characteristics are the three main influencing factors that affect drought, and these three factors work together to influence the spatial and temporal distribution patterns of drought, which in turn affects drought evolution^7–9^. Water regime characteristics reflect the subsurface factors which affect the evolution of drought and influence the lag and attenuation characteristics of drought propagation^10^. Climate change acts directly on the spatial and temporal patterns of drought by altering the spatial and temporal distribution of meteorological elements such as precipitation and temperature^11^. Human activities, such as land use and reservoir construction, can change drought evolution characteristics and processes, and land use change is one of the main influencing factors of drought evolution^12^. Human activities change the subsurface factors which act on the spatial pattern of drought by changing land use patterns, which in turn have a significant impact on drought^13^.

From 1980 to 2010, the land use type of Loess Plateau in Shaanxi has changed a lot due to the reform and opening up and the project of returning farmland to forest. China began to carry out the major measures of reform and opening up in the late 1970s. The level of industrialization has been raised by big percentages, urbanization has sped up, and the categories of land use have changed significantly. At the same time, it also has a certain impact on climate change, with intensified climate warming and frequent occurrence of extreme climates. Since the late 1990s, drought disasters have become increasingly frequent and severe throughout China due to the effects of increased global warming and the continued weakening of the East Asian summer winds, especially in parts of northern China, with interannual major droughts in 1995, 1997, and 2000 in the eastern part of the northwestern region and in northern China^14,15^. The Loess Plateau is located in the eastern part of the northwest region of China, with severe soil erosion and frequent droughts, which have a great impact on local socio-economic and water resources management^16^. To change this situation, in the late 1990s, the project of returning farmland to the forest was first carried out in Shaanxi, Sichuan and Gansu provinces. Since then, the type of land use in Shaanxi has changed considerably. From 1999 to 2008, China has finished 403 million mu (a Chinese unit of area, it is equal to 1/15 of a hectare or 1/6 an acre) of farmland to forest totally, of which 139 million mu of arable lands were turned into forests, 237 million mu of unused areas were turned into forests, and 27 million mu of mountains were closed for being turned into forests^17^.

Many scholars have conducted studies related to drought in northwest China, but such studies only focus on the effects of meteorological factors such as precipitation and temperature on drought. Kong et al. used the precipitation and soil moisture data to calculate SPI (standardized precipitation index) and SSMI (standardized soil moisture index), and used the run-length theory to identify drought characteristic variables, and studied the drought changes in Yulin City^18^. Wang et al. calculated SPI and SPEI of different scales to analyze characteristics of drought variation with time and space on the Loess Plateau in the past 57 years, and used cross wavelet transform to discuss the correlation analysis between drought index and atmospheric circulation^19^. Liu et al. established aridity index AI and concluded that precipitation and actual water vapor pressure are the main climatic factors affecting AI changes in Gansu, Ningxia, Qinghai, and Shaanxi, and the main climatic factors affecting AI changes in Xinjiang are potential evapotranspiration, solar Radiation and mean air temperature^20^. Droughts are not just caused by climate change. Different from the above research views, this paper studies its impact on drought from the perspective of land-use types.

In summary, among the factors affecting drought, the indirect role of human activities on drought cannot be ignored. From 1980 to 2010, human activities such as reform and opening up and the project of returning farmland to forest have changed the land use type of the Loess Plateau in Shaanxi. To study the effects of land use type changes on drought in the study area, the following experiments were conducted in this paper based on the method of univariate linear regression. Firstly, based on the meteorological and land use data of the study area from 1980 to 2010, eight stations with different land use types were selected. Then the drought index SPI was calculated for the eight stations, and the response of SPI index to different land use types was analyzed. Considering the large time span, this performance was clearly described according to the phases 1980–1990, 1990–1995, 1995–2000, 2000–2005, and 2005–2010 based on the obtained remote sensing data, so as to visualize the changes of drought in more detail with six phases.

## Materials and methods

### Study area

**Figure 1:**
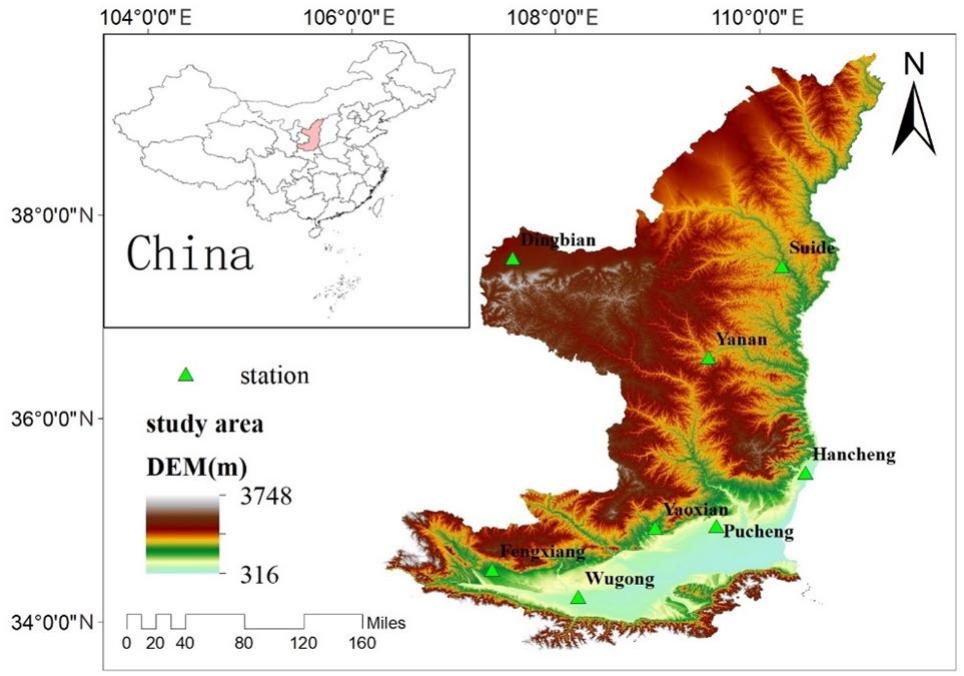
Location of the study area (Arcmap10.8 https://www.resdc.cn/).

Shaanxi Loess Plateau is situated at 33° 41′ 35′′ north latitude and 106° 19′ 14′′ east longitude, including Yulin, Yan’an, Tongchuan, Xianyang, Xi’an, Weinan, Yangling and parts of Baoji City. Its area of about 130,000 square kilometers. Northern Shaanxi is situated in the center of the Loess Plateau. The height is 900–1600 m above sea level. The height of the terrain decreases from northwest to southeast. It has the semi-arid temperate continental monsoon climate zone. It is dry and rainy, extremely lack of water resources, and the temporal distribution of temperature and precipitation is different greatly. The annual average precipitation is about 300 mm, and the uneven spatial distribution is opposite to terrain. The Guanzhong part belongs to the southern part of the Loess Plateau and is located in the Weihe Plain, with an altitude of 325–900 m. The annual average temperature is above 12 °C, and the annual average precipitation is 600–700 mm. The geographical location of the study area is shown in Fig. 1.

### Data

In this study, the data used include Digital Elevation Model (DEM) data, land use data and meteorological data. The DEM and land use type data of the study area were obtained under the crop of ArcGIS. The meteorological data were collated by Matlab fitting.

The DEM data are from the Resource and Environmental Science and Data Center of the Chinese Academy of Sciences (https://www.resdc.cn/). The spatial distribution data of China’s altitude (DEM) in this website comes from the radar topography mapping SRTM (Shuttle Radar Topography Mission, SRTM) data of the US space shuttle Endeavour. SRTM data has the advantages of strong realism and free access. Many applied studies around the world use SRTM data to carry out the environmental analysis. The data set is 90 m provincial data generated by sorting and splicing based on the latest SRTM V4.1 data.

**Figure 2:**
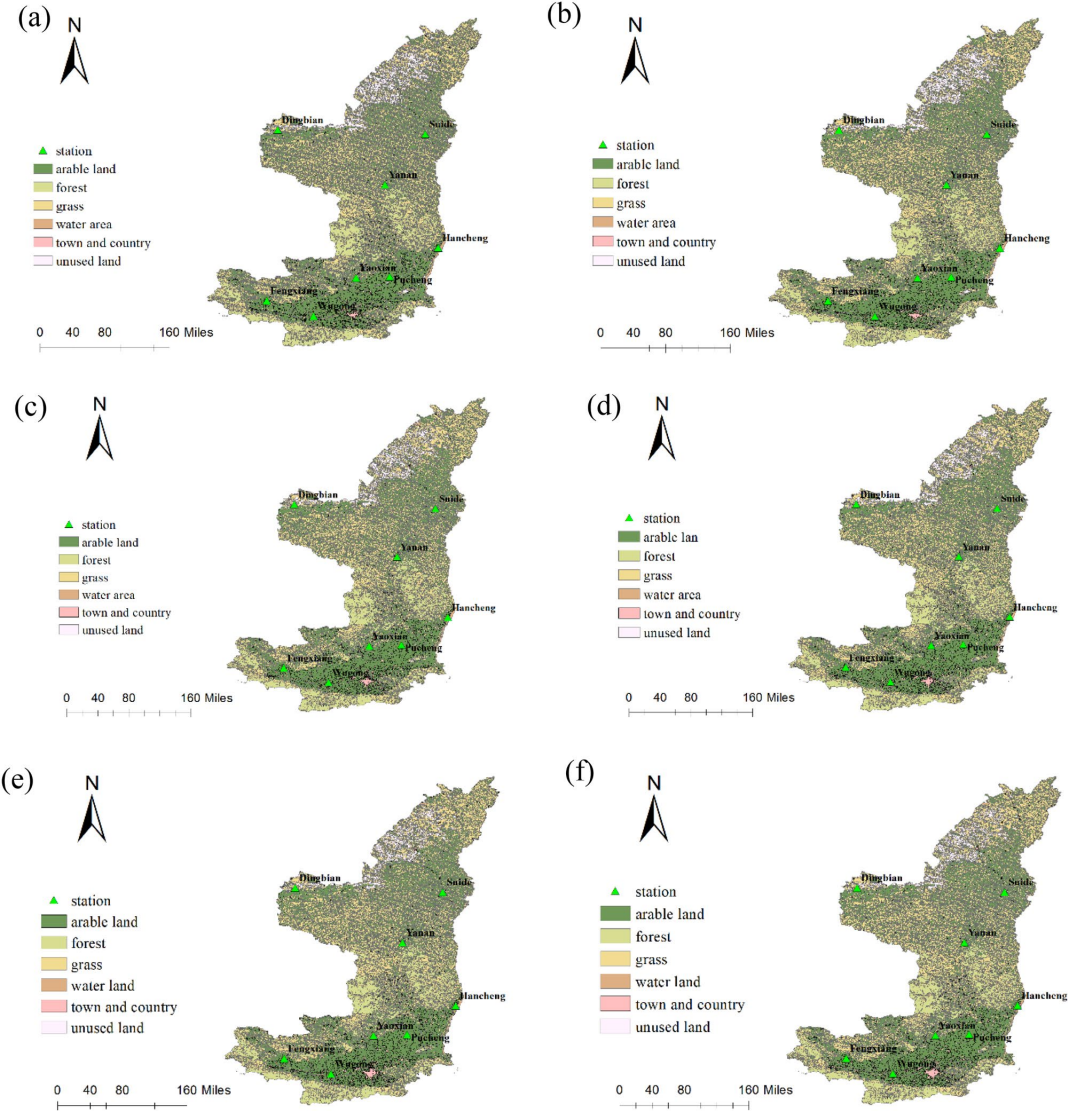
Land use of study area in 1980 (**a**), 1990 (**b**), 1995 (**c**), 2000 (**d**), 2005 (**e**), 2010 (**f**) (Arcmap10.8 https://www.resdc.cn/).

The land use data comes from the Resource and Environmental Science and Data Center of the Chinese Academy of Sciences (https://www.resdc.cn/). The years 1980, 1990, 1995, 2000, 2005 and 2010 with a resolution of 1 km of land use data are used in this paper. After reclassification, there are 6 categories of land use of forest, arable land, unused area, grass, water area, and town and country. The categories of land use each year are shown in Fig. 2.

The basic meteorological data is from the China Meteorological Data Network (http://data.cma.cn/). The daily data of the average temperature and precipitation of 8 meteorological stations in the Loess Plateau region of Shaanxi from 1980 to 2010 were obtained. These data are carefully filtered to get rid of unusual data, and next the cubic spline interpolation method is used to interpolate the individual missing measurement values. The geographic locations of meteorological stations and the types of land use in each year are shown in Table 1.

**Table 1:** Information of stations.

Station number	Name	Latitude	Longitude	Height	1980	1990	1995	2000	2005	2010
53725	Dingbian	37.58	107.58	1361.3	Arable land	Arable land	Arable land	Arable land	Town and country	Town and country
53754	Suide	37.5	110.22	928.5	Forest	Forest	Forest	Forest	Forest	Forest
53845	Yanan	36.6	109.5	958.8	Forest	Forest	Forest	Forest	Forest	Forest
53948	Pucheng	34.95	109.58	499.9	Arable land	Arable land	Grass	Town and country	Town and country	Town and country
53955	Hancheng	35.47	110.45	458.8	Arable land	Arable land	Arable land	Arable land	Arable land	Town and country
57025	Fengxiang	34.52	107.38	781.1	Town and country	Town and country	Grass	Town and country	Town and country	Town and country

### Methodology

#### Standardized precipitation index

The standardized precipitation index (SPI) was put forward by McKee et al. in 1993^21^. It is a common analytical method in precipitation analysis and drought monitoring, and has been extensive applied in meteorological drought monitoring^22,23^. The following are the specific calculation steps.

The probability density function of the $\Gamma$-distribution of the monthly precipitation x:

1 $${z}_{i}={\frac{6}{{c}_{s}} \left(\frac{{c}_{s}}{2}{\varphi}_{i}+1\right)}^\frac{1}{3}-\frac{6}{{c}_{s}}+\frac{{c}_{s}}{6},$$

2 $$\Gamma \left(\upgamma \right)={\int}_{0}^{\infty }{x}^{\gamma -1}{e}^{-x}dx.$$

$\beta > 0,\;\Upsilon > 0$ are scale and form parameters, respectively, $\beta$ and $\Upsilon$ can be obtained by the maximum likelihood estimation method:

3 $$\widehat{\gamma }=\frac{1+\sqrt{1+4A/3}}{4A},$$

4 $$\widehat{\beta }=\overline{x }/\widehat{\gamma ,}$$

5 $$\mathrm{A}=\mathrm{lg}\widehat{x}-\frac{1}{n}\sum_{i=1}^{n}lg{x}_{i}.$$

For the precipitation *x*_0_ in a certain year, the probability that the random variable *x* is less than the event *x*_0_ can be calculated as:

6 $$\mathrm{P}\left(\mathrm{x}<{x}_{0}\right)={\int}_{0}^{\infty }f\left(x\right)dx.$$

Using numerical integration, an estimate of event probability can be calculated by substituting (1) into (6).

The above formula does not take the case into account where the precipitation is 0. In reality, the situation where the precipitation is 0 still exists. The probability of an event with zero precipitation is estimated by:

7 $${\text{P}}\left( {{\text{x}} = 0} \right) = {\text{m}}/{\text{n}}.$$

where m is the number of samples with 0 precipitation, and n is the total number of samples.

Normalize the probability of $\Gamma$ distribution and solve for the SPI value:

8 $$\mathrm{SPI}=\mathrm{S}\frac{t-\left({c}_{2}t+{c}_{1}\right)t+{c}_{0}}{\left(\left({d}_{3}t+{d}_{2}\right)t+{d}_{1}\right)t+1},$$

$$\mathrm{t}=\sqrt{ln\frac{1}{{P}^{2}}}$$, when P > 0.5, P = 1.0 − P, S = 1; when P ≤ 0.5, S =  − 1, *c*_0_ = 2.515517, *c*_1_ = 0.802853, *c*_2_ = 0.012328, *d*_1_ = 1.432788, *d*_2_ = 0.189269, *d*_3_ = 0.001308.

#### Univariate linear regression

Linear regression is mainly used to describe the linear relationship between the dependent variable y and the independent variable x. The univariate linear regression method is to analyze the relationship between several data point sets (x_i_, y_i_) (i = 1, 2,…, n), and draw up a linear regression equation between variables x and y. Its basic formula is:

9 $${y}_{i}=A{x}_{i}+B\left(i=\mathrm{1,2},\ldots ,n\right).$$

#### Land use type transfer matrix

The land use transfer matrix can reflect the change of the land use structure in all aspects and show how the land use changes^24^. It originates from the quantitative description of system state and state transition in system analysis, and represents the procedure of system transition from time S to time S + 1 in a certain period. It can effectively reflect the temporal and spatial evolution of land use patterns^25^, and its formula is:

10 $${s}_{ij}=\left[\begin{array}{cc}\begin{array}{cc}{S}_{11}& {S}_{12}\\ {S}_{21}& {S}_{22}\end{array}& \begin{array}{cc}\cdots & {S}_{1n}\\ \cdots & {S}_{2n}\end{array}\\ \begin{array}{cc}\cdots & \cdots \\ {S}_{n1}& {S}_{n2}\end{array}& \begin{array}{cc}\cdots & \cdots \\ \cdots & {S}_{nn}\end{array}\end{array}\right].$$

In the formula: S_ij_ is the land-use status at the beginning and end of the period; n is the number of land-use types.

At present, the vector in the commonly used land-use state transition matrix can be the area of land use type, or the probability of transition from original land-use type to the final land-use type, which is called the Markov transition probability matrix^26^. In this study, the area of each land use type was counted using ArcGIS, and then the transfer analysis of land use types in the study area was performed.

## Results and analysis

### From 1980 to 1990

From the land use type transfer matrix (Table 2), a complex interconversion of land use types occurred in the study area. The area of arable land, water area, and unused land decreased by 0.02%, 3.54%, and 1.89%, respectively. Areas of forest, grass and town and country increased by 0.13%, 0.17% and 3.56% respectively. The water area decreased the most, mainly transferred to arable land, while the town and country increased the most at this time, and the main source of transfer was arable land. This is due to the fact that China’s economy began to develop at this time and urbanization began to accelerate.

**Table 2:** Transfer matrix of land use types in the study area from 1980 to 1990 (km^2^).

1980	1990					
	Grass	Town and country	Arable land	Forest	Water area	Unused area
Grass	42,201.22051	2.557753	2708.469982	411.397571	35.814914	262.164944
Town and country	3.513511	1778.674357	516.939021	1.105836		0.376887
Arable land	2360.700571	153.468322	50,263.53377	189.814082	50.716512	95.135768
Forest	441.991315	4.801844	282.502519	19,671.1085	2.092333	11.382358
Water area	74.129365	0.061531	142.959443	8.376883	1363.987946	7.21326
Unused area	186.94696	4.715451	110.9011	15.545843	9.464633	5749.66462

**Figure 3:**
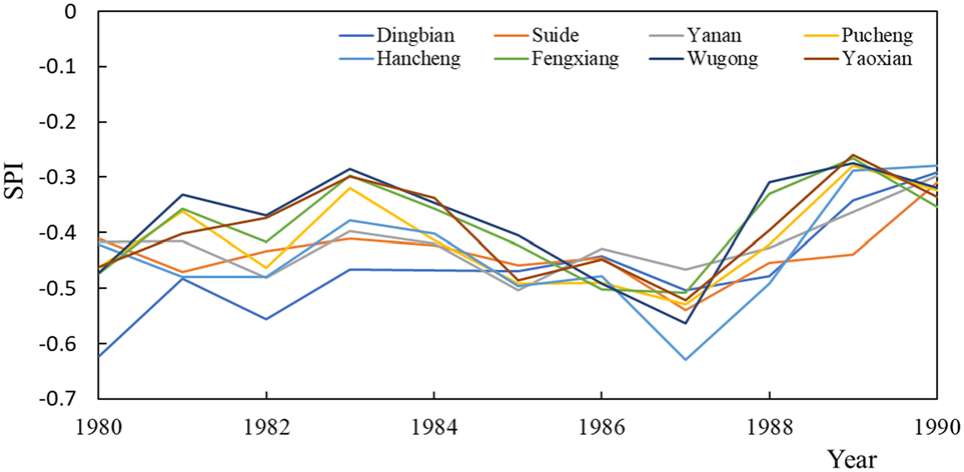
SPI changes at each site from 1980 to 1990.

From 1980 to 1990, the land use types of the eight study stations did not change, the SPI values increased, and the intensity of drought decreased (Fig. 3). Taking the SPI for each use type of site as the mean value, the SPI value in 1980 was − 0.42773 for forest, − 0.48812 for arable land, and − 0.47317 for town and country. In 1990, the SPI of the forest is − 0.30283, the SPI value of arable land is − 0.31011, and the SPI value of town and country is − 0.35354. By observing the change of SPI of each land-use type, the SPI of arable land has the largest change with a rate of change of 0.00902 year^−1^, while the forest type has the smallest change with a rate of change of 0.005051 year^−1^. In this stage, arable land has the greatest impact on drought because crops are affected by climatic conditions. Although the forest has the least impact on drought, overall, the drought intensity of forest land is relatively high.

### From 1990 to 1995

From 1990 to 1995, according to the land use type transition matrix (Table 3), only the unused land type decreased by 19.87%, mainly to grass, followed by arable land. The areas of arable land, forest, grass, water area and town and country all increased by 0.27%, 0.60%, 1.64%, 0.39% and 6.29% respectively. The largest increase is in town and country, and the main source of transfer of it is arable land. Similar to the situation in the 1980s, China’s urbanization was rapid and unused land was developed to provide for China’s industrialization.

**Table 3:** Transfer matrix of land use types in the study area from 1990 to 1995 (km^2^).

1990	1995					
	Grass	Town and country	Arable land	Forest	Water area	Unused area
Grass	44,185.77516	6.635969	656.486777	231.971975	53.569883	133.6654
Town and country	16.711513	1811.509578	111.51209	2.780686	1.796169	
Arable land	384.005471	256.80545	53,126.6207	134.428586	68.879502	49.567196
Forest	233.240712	10.88188	85.898481	19,967.26462	5.636921	13.107064
Water area	39.578843	0.589351	86.454135	3.107755	1321.739618	3.619636
Unused area	1247.218077	2	140.818456	64.488865	13.785722	4653.807856

**Figure 4:**
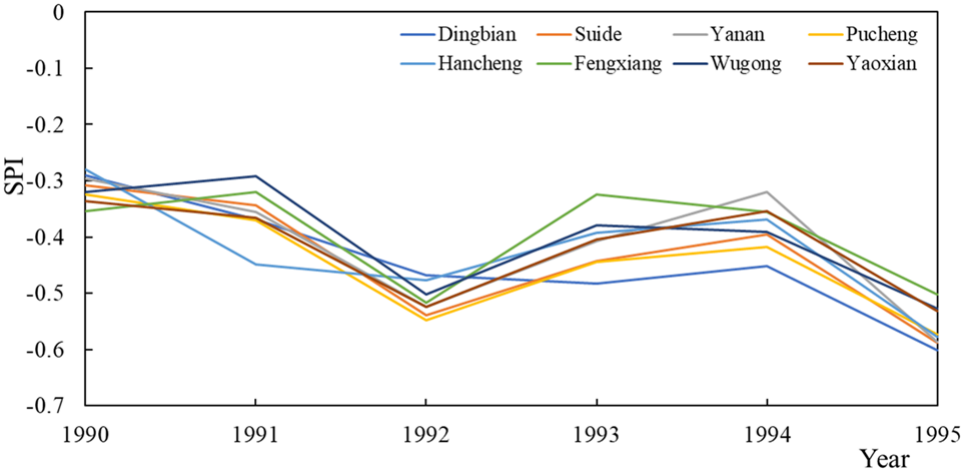
SPI changes at each site from 1990 to 1995.

From1990 to 1995, the SPI values all showed a downward trend, and the drought intensity increased (Fig. 4). In 1990, the SPI value of forest was the largest, which was − 0.30283, and the SPI value of town and country was the smallest, which was − 0.35354. In 1995, the SPI value of grass was the largest, which was − 0.53743, and the SPI value of forest was the smallest, which was − 0.58723. In Pucheng and Fengxiang, changes in land use types occurred during this period. Pucheng changed from arable land to grass, and Fengxiang changed from town and country to grass. The change rate of SPI in Pucheng was − 0.0367 year^−1^, and the change rate of SPI in Fengxiang was − 0.0187 year^−1^, indicating that the change from town and country to grass had less impact on SPI. The change in the substrate from town and countryside to grass has led to an increase in vegetation and a reduction in the urban heat island effect. Compared with other stations with unchanged land use types, the changes in SPI values are all larger than those with changes in land use. Among them, the forest land type station has the smallest SPI value change rate, which is − 0.0402 year^−1^. Similar to the previous stage, the response of the drought index to the arable land is greater than that of the forest. The drought intensity of the forest land is still the largest, followed by the arable land, and finally the town and country. At this time, the disturbance of arable land by human activities is less than that of town and country.

### From 1995 to 2000

From 1995 to 2000, according to the land use type transition matrix (Table 4), arable land, grass and water area all decreased by 0.05%, 0.37% and 3.53% respectively. Forest land, town and country and unused land increased by 0.41%, 6.16% and 0.13% respectively. The largest reduction was in water area, and its main transfer out types were arable land and grass. Influenced by the rapid development of economic construction and urbanization, the area of town and country has increased relatively quickly, and the main types of transfer are arable land and grassland.

**Table 4:** Transfer matrix of land use types in the study area from 1995 to 2000 (km^2^).

1995	2000					
	Grass	Town and country	Arable land	Forest	Water area	Unused area
Grass	45,689.25741	22.925261	246.151783	78.586824	20.021979	59.611618
Town and country	0.185498	2067.675398	19.934811	0.549288	0.077234	
Arable land	154.05456	130.274158	53,800.00563	41.393344	34.722952	42.206942
Forest	18.433184	4.009388	54.016233	20,351.89242	2.030777	0.707807
Water area	36.094969	1.765388	58.585737	2.027309	1360.916083	1.25
Unused area	52.760802		16.482095	20.660092	0.640381	4761.904691

**Figure 5:**
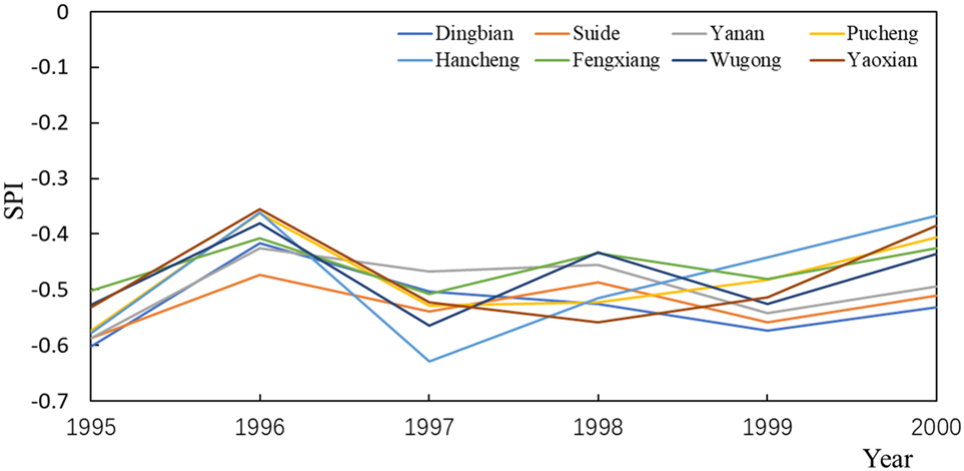
SPI changes at each site from 1995 to 2000.

During the period, according to Fig. 5, the SPI showed a roughly increasing trend. In 1997, a severe drought occurred, and the SPI decreased. In 1995, the SPI value of grass was the largest, which was − 0.53743, and the SPI value of forest was the smallest, which was − 0.58723. In 2000, the SPI value of forest was still the smallest, which was − 0.50254. The SPI value of town and country is the largest at − 0.41615. Comparing the change speed of each land-use type, the forest has the smallest change rate, which is 0.0043 year^−1^. The change rate of arable land is the largest, which is 0.01234 year^−1^. During this period, both Pucheng and Fengxiang were urbanized, and the grass areas were converted into town and country areas. The average rate of change for the two sites was 0.01028 year^−1^, which is a small rate of change. The impact of the land use type shift on the arid climate is mitigated by the extensive greening of the city and the lesser degree of grassland development. This stage is still the same as the previous stage, the drought intensity of forest is the largest, but the impact on drought is the least. Arable land has a greater impact on drought, but the intensity of drought is bigger than that of forest and greater than that of town and country.

### From 2000 to 2005

From 2000 to 2005, according to Table 5, arable land and unused land decreased by 2.58% and 0.75% respectively. Areas of forest, grass, water area and town and country all increased by 4.94%, 0.09%, 4.46% and 10.30% respectively. In the late 1990s, the project of returning farmland to the forest was implemented in Shaanxi, and the reduction rate of arable land was greatly increased, and the main types of conversion were forest and grass. At the same time, the area of forest and grass has also greatly increased due to the beginning of the project of returning farmland to the forest. According to Table 5, the main transfer type is also arable land. At the same time, in the early twenty-first century, China’s society developed rapidly, the urbanization process accelerated, and the main type of transfer was arable land.

**Table 5:** Transfer matrix of land use types in the study area from 2000 to 2005 (km^2^).

2000	2005					
	Grass	Town and country	Arable land	Forest	Water area	Unused area
Grass	45,097.39842	20.897168	300.612855	468.292252	18.546955	45.762937
Town and country	2.780769	2199.288821	23.089699	0.421173	1.038354	0.030777
Arable land	814.322533	229.183372	52,447.5511	610.294628	90.507136	4.855583
Forest	46.1498	16.170546	19.39619	20,421.30682	4.120245	12.26055
Water area	3.092748	4.482719	41.735879	1.072158	1364.962338	
Unused area	57.078922	4.030781	25.908805	9.342373	3	4767.258816

**Figure 6:**
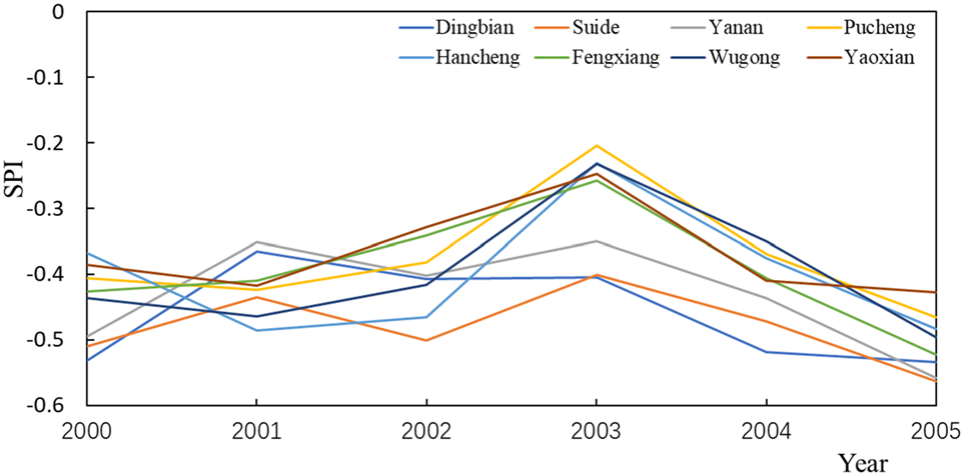
SPI changes at each site from 2000 to 2005.

During the period, the SPI value first increased, and then began to decrease after reaching a peak in 2003 (Fig. 6). Forest had the smallest SPI value in 2000 at − 0.50254. The SPI value of town and country is the largest at − 0.41615. In 2005, the SPI value of forest land is still the smallest at − 0.56044. The SPI value of arable land is the largest, which is − 0.46885. During this period, the land use type in Dingbian changed, and there was a change from arable land to town and country. The change of land use type has a greater impact on SPI, which is − 0.01343 year^−1^. For other stations with the same land use type, the rate of change of SPI is smaller than that of Dingbian. The minimum rate of change for the arable land was − 0.0035 year^−1^. The rate of change was greatest in the forest at − 0.01137 year^−1^. From this stage, the response relationship of the drought index to land use has changed. The drought intensity of forest is still the largest, but the drought intensity of town and country is greater than that of arable land. The response of forest land to drought becomes the largest, followed by town and country and finally arable land. The reason may be that the disturbance intensity of arable land has been greatly increased by human activities.

### From 2005 to 2010

From 2005 to 2010, according to Table 6, the area of arable land, water area and unused land decreased by 0.33%, 2.29% and 1.87% respectively. With the deepening of the returning farmland to forest, the area of forest land, grassland and town continued to increase, increasing by 0.98%, 0.01% and 2.73% respectively. The largest reduction was in water lands. The main type of transfer out of water lands is arable land, while the type of transfer out of unused land is grass. The increase in town and country remains at a high rate, with the main type of conversion being arable land. Arable land occupies a large proportion of the complex conversion of land use types.

**Table 6:** Transfer matrix of land use types in the study area from 2005 to 2010 (km^2^).

2005	2010					
	Grass	Town and country	Arable land	Forest	Water area	Unused area
Grass	45,794.26641	9.700376	83.512163	112.528167	7.728545	9.087991
Town and country	0.18628	2464.295359	9.492134	0.053636	0.025998	
Arable land	115.281667	68.294851	52,515.0542	103.44604	47.328995	7.923942
Forest	12.509451	5.038267	10.169067	21,478.49265	1	0.056775
Water area	14.867528	1.030777	63.374712	5.746214	1388.292803	1.671161
Unused area	91.860299	3	16.253978	1.257804	0.824754	4715.971828

**Figure 7:**
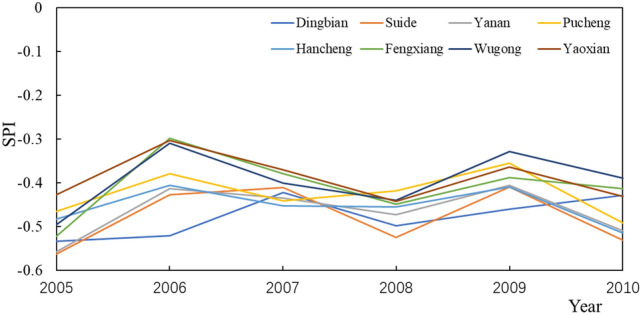
SPI changes at each site from 2005 to 2010.

During the period, the change of SPI generally showed a stable trend with little change (Fig. 7). Forest land had the smallest SPI value in 2005 at − 0.56044. The SPI value of arable land is the largest, which is − 0.46885. In 2010, the SPI value of forest land is still the smallest, which is − 0.51991. The SPI value of arable land is the largest at − 0.41049, followed by town and country, with an SPI value of − 0.46269. During this stage, the land use type of Hancheng changed from arable land to town and country. In the case of changes in land use types, compared with other sites with no changes in land use types, Hancheng has a larger change rate of − 0.0048, and is the only site with a decreasing SPI value. The change rate of arable land is the smallest, which is 0.002225. The forest land had the largest rate of change, 0.00475. This stage is the same as the previous stage, the drought intensity of forest is still the largest, but the drought intensity of town and country is greater than that of arable land. Forest had the greatest response to drought, followed by town and country, and finally arable land.

## Discussion

The change of land use type in each stage represents the difference of ecological environment in each stage, and the impact on meteorological drought is also different^26^. From the aspect of different land-use types, the response relationship of drought index SPI to different land-use types is analyzed in this paper. Since the land use types of the stations in this study only involve three types of arable land, forest, and town and country, only the SPI values of these three types are sorted.

According to the above analysis, it can be seen that the outcomes of this study show that the law is divided into two stages which are the twentieth century and the twenty-first century. From the perspective of drought intensity, from 1980 to 2010, the drought intensity of forest has always been the largest. The vegetation of the forest is less disturbed by humans and is sensitive to climatic conditions. Under the local dry and rainy climate conditions, the drought intensity is relatively large, while the drought intensity of the arable land and town and country are relatively small. However, in the twentieth century, the intensity of aridity of arable land was greater than that of town and country. In the twenty-first century, it was less than that of town and country. This is because with the continuous advancement of farming technology, the disturbance of human activities, such as water-saving irrigation technology, has improved water use efficiency and alleviated the drought of arable land^27^.

In the same period, the changing trend of SPI is roughly the same, but the change degree is different, indicating that different land uses have different degrees of adjustment to climate. The drought had the weakest response to the forest in the twentieth century and had the least impact on SPI. This is because forest species have deep root systems, and under severe drought conditions, they can use the water stored in the deep soil to alleviate changes in arid climates^28^. However, due to the time lag effect of deep soil moisture changes with precipitation, the response of forest species to drought may be delayed^29^. Therefore, after the twenty-first century, with the project of returning farmland to forest moving forward, the forest area has increased substantially, and the impact of forest land on drought has become the largest. During the twentieth century, drought had the greatest degree of response to arable land. Crops on arable land are restricted by natural conditions such as rainfall and temperature, so they have a strong response to drought. From 2000 to 2010, arable land had the least response to drought. Except for the natural climatic conditions, the possible reason is that the arable area is more affected by irrigation and artificial water diversion, so the SPI cannot fully reflect the degree of surface aridity^30^. After the twenty-first century, the drought intensity of town and country is greater than that of arable land, and the degree of influence on the drought index is also greater than that of arable land. This is because with the in-depth development of urbanization, the urban heat island effect increases, and the intensity of drought also increases, which also has influence on the change of drought^31^.

Land use is the result of the connective effect of nature and humanity. Climate change affects the growth of vegetation, and the mutual conversion of unused land, grass and forest land occurs. In addition, due to economic development, the type of land use has been transformed from arable land to town and country due to human action^32^. Changes in several different land-use types also have large or small impacts on drought. Both arable land and town and country are disturbed by human activities, but the greening construction inside the city has been improved^33^. The rate of change in the same period is compared. Therefore it can be seen that the transition from arable land to grass and the change from arable land to town and country have a greater impact on drought, while the transition from town to grass and grass to town has less impact on drought.

In summary, types of land use mainly affect the response relationship of drought to them through the degree of vegetation coverage and the disturbance of human activities. Therefore, while monitoring the local drought, we should not only attach importance to the local climate change, but also attach importance to the impact of human activities. In this study, different types of land use may cancel each other out and then affect the average value of the drought index. For example, arable land is converted into the grass, and some grasslands are reclaimed into arable land. The impact of land-use change on drought index needs to be further studied in depth and detail.

## Conclusion

1. From 1980 to 2000, the drought intensity of various land-use types was: forest > arable land > town and country. From 2000 to 2010, the drought intensity of various land-use types was: forest > town and country > arable land.
2. The response degree of drought index to land use types is different. From 1980 to 2000, arable land had the greatest response to drought index, followed by town and country, and forest had the weakest response to drought index. In the twenty-first century, the forest has the strongest response to drought index, followed by town and country, and finally arable land.
3. The area of forest, grass and town and country is increasing continuously, while the area of arable land is decreasing year by year, and the types of land use are constantly undergoing complex changes. The drought index has a strong response to the change of arable land to grass and arable land to town and country, but has a weak response to the mutual transformation of grass and town and country.

## Data Availability

The data that support the findings of this study are available from the Resource and Environmental Science and Data Center of the Chinese Academy of Sciences but restrictions apply to the availability of these data, which were used under license for the current study, and so are not publicly available. Data are however available from the authors upon reasonable request and with permission of the Resource and Environmental Science and Data Center of the Chinese Academy of Sciences.

## References

[1] Qiang Z, Yubi Y, Yaohui L, Zhexian L, Cunjie Z, Dongliang L (2015). Research progress of drought meteorological disaster monitoring and early warning and mitigation technology in northwest China and its outlook. Adv. Earth Sci..

[2] Wu J, Chen X, Yao H (2017). Non-linear relationship of hydrological drought responding to meteorological drought and impact of a large reservoir. J. Hydrol..

[3] Wang J, Guo J, Zhou Y (2007). Progress and prospects of drought indicator research. Geogr. Arid Reg..

[4] Mckee, T. B., Doesken, N. J. & Kleist, J. The relationship of drought frequency and duration of time scales. In *Eighth Conference on Applied Climatology* 179–186 (American Meteorological Society, 1993).

[5] Chen LL, Liu PX, Yao YL, Zhu XJ, Zhao ML (2013). Annual and spring variation characteristics of SPI and Z index in different climatic zones of Gansu Province from 1960 to 2010. J. Ecol..

[6] Zhihui S, Zhiliang W, Xuemei C, Qiong Y, Zhichao L, Yanpeng L (2013). Characteristics of drought changes in the Loess Plateau region of Shaanxi Province from 1971 to 2010 based on standardized precipitation index. China Desert.

[7] Konapala G, Mishra A (2017). Review of complex networks application in hydroclimatic extremes with an implementation to characterize spatio-temporal drought propagation in continental USA. J. Hydrol..

[8] Apurv T, Sivapalan M, Cai XM (2017). Understanding the role of climate characteristics in drought propagation. Water Resour. Res..

[9] Jehanzaib M, Kim TW (2020). Exploring the influence of climate change-induced drought propagation on wetlands. Ecol. Eng..

[10] Wu JF, Chen XW, Love CA (2020). Determination of water required to recover from hydrological drought: Perspective from drought propagation and non-standardized indices. J. Hydrol..

[11] Jehanzaib M, Sattar MN, Lee JH (2020). Investigating effect of climate change on drought propagation from meteorological to hydrological drought using multi-model ensemble projections. Stoch. Environ. Res. Risk Assess..

[12] Zhou JJ, Li QQ, Wang LY (2019). Impact of climate change and land-use on the propagation from meteorological drought to hydrological drought in the eastern Qilian Mountains. Water.

[13] Xu Y, Zhang X, Wang X (2019). Propagation from meteorological drought to hydrological drought under the impact of human activities: A case study in northern China. J. Hydrol..

[14] Qian ZA, Song MH, Wu TW, Cai Y (2017). Review of world arid climate research dynamics and progress (II): Major research progress. Highl. Meteorol..

[15] Yang R, Geng G, Zhou H, Wang T (2021). Spatial and temporal evolution characteristics of meteorological drought in the Weihe River basin based on SPEI___PM index. China Agric. Meteorol..

[16] Shuo W, Chenfeng C, Qin D (2021). Contributions of vegetation greening and climate change to evapotranspiration trend after large-scale vegetation restoration on the Loess Plateau. China. Water.

[17] Nazarbakhsh M, Ireson AM, Barr AG (2020). Controls on evapotranspiration from jack pine forests in the Boreal Plains Ecozone. Hydrol. Process..

[18] Kong G, Shanliang Z, Lu W, Shengzhi H, Jueying B (2021). Analysis of spatial and temporal evolution of drought characteristics of different classes in Yulin City. People’s Pearl River.

[19] Wang J-R, Sun C-J, Zheng Z-J, Li X-M (2021). Drought characteristics of the Loess Plateau in the last 57 years and its relationship with atmospheric circulation. J. Ecol..

[20] Liu LIL, Han L, Han YG, Gao Y, Peng L (2021). Spatial and temporal variation of dryness index and its response to climate factors in Northwest China from 1989–2019. J. Appl. Ecol..

[21] Liu C, Chen H, Chen H, Chen N, Ding YJ (2018). SPI-based analysis of drought characteristics in Shaanxi Province from 1961 to 2016. Jiangxi J. Agric..

[22] Wang X, Hu C, Wei W, Yu Y (2016). SPI-based spatial and temporal characteristics of drought in the Weibei Loess Plateau. J. Ecol. Environ..

[23] Wang, C.-W. *et al*. Effects of climate and land use changes on the changes of drought index in Wuding River Basin from 1982 to 2010. *Hydrology* 17–23.

[24] Xiong Y, Chen C (2008). A review of research on land use and land cover change[J]. J. Langfang Normal University: Natural Science Edition..

[25] Cheng L, Xu Z, Luo R (2009). Spatial-temporal characteristics of lucc and driving factor analysis for the Wei River basin from 1980 to 2000. Res. Soil Water Conserv..

[26] Yanju L, Jianli D, Junyong Z (2019). Response of vegetation cover to drought on the northern slopes of Tianshan Mountain from 2001–2015—Based on land use/land cover analysis. J. Ecol..

[27] Deng XP, Shan L, Zhang HP, Turner NC (2006). Improving agricultural water use efficiency in arid and semiarid areas of China. Agric. Water Manage..

[28] Davidson EA, Verchot LV, Cattnio JH, Ackerman IL, Carvalho JEM (2000). Effects of soil water content on soil respiration in forests and cattle pastures of eastern Amazonia. Biogeochemistry.

[29] Anderegg WRL, Schwalm C, Biondi F, Camarero JJ, Koch G, Litvak M, Ogle K, Shaw JD, Shevliakova E, Williams AP, Wolf A, Ziaco E, Pacala S (2015). Pervasive drought legacies in forest ecosystems and their implications for carbon cycle models. Science.

[30] Gao ZQ, Liu JY (2006). Response of LUCC to climate change in China from 1980 to 2000. J. Geogr..

[31] Jiang WJ, Zhao HM, Tang HH (2017). Study on the relationship between surface drought and urban surface heat island in Poyang Lake area. J. Huazhong Normal Univ. (Nat. Sci. Ed.).

[32] Shuyi Z, Zhaoning G, Xuying L (2015). Correlation analysis of vegetation cover and drought conditions in North China from 2001 to 2013. J. Geogr..

[33] Yang J, Huang CH, Zhang ZY, Wang L (2014). The temporal trend of urban green coverage in major Chinese cities between 1990 and 2010. Urban For. Urban Green..

